# Dystroglycan Binding to α-Neurexin Competes with Neurexophilin-1 and Neuroligin in the Brain*

**DOI:** 10.1074/jbc.M114.595413

**Published:** 2014-08-25

**Authors:** Carsten Reissner, Johanna Stahn, Dorothee Breuer, Martin Klose, Gottfried Pohlentz, Michael Mormann, Markus Missler

**Affiliations:** From the ‡ Institute of Anatomy and Molecular Neurobiology, Westfälische Wilhelms-University, Vesaliusweg 2-4, 48149 Münster, Germany,; § Institute of Medical Physics and Biophysics, Westfälische Wilhelms-University, Robert-Koch Strasse 31, 48149 Münster, Germany, and; ¶ Cluster of Excellence EXC 1003, Cells in Motion, 48149 Münster, Germany

**Keywords:** Adhesion, Autism, Disulfide, Glycomics, Molecular Modeling, Site-directed Mutagenesis

## Abstract

α-Neurexins (α-Nrxn) are mostly presynaptic cell surface molecules essential for neurotransmission that are linked to neuro-developmental disorders as autism or schizophrenia. Several interaction partners of α-Nrxn are identified that depend on alternative splicing, including neuroligins (Nlgn) and dystroglycan (αDAG). The trans-synaptic complex with Nlgn1 was extensively characterized and shown to partially mediate α-Nrxn function. However, the interactions of α-Nrxn with αDAG, neurexophilins (Nxph1) and Nlgn2, ligands that occur specifically at inhibitory synapses, are incompletely understood. Using site-directed mutagenesis, we demonstrate the exact binding epitopes of αDAG and Nxph1 on Nrxn1α and show that their binding is mutually exclusive. Identification of an unusual cysteine bridge pattern and complex type glycans in Nxph1 ensure binding to the second laminin/neurexin/sex hormone binding (LNS2) domain of Nrxn1α, but this association does not interfere with Nlgn binding at LNS6. αDAG, in contrast, interacts with both LNS2 and LNS6 domains without inserts in splice sites SS#2 or SS#4 mostly via LARGE (like-acetylglucosaminyltransferase)-dependent glycans attached to the mucin region. Unexpectedly, binding of αDAG at LNS2 prevents interaction of Nlgn at LNS6 with or without splice insert in SS#4, presumably by sterically hindering each other in the u-form conformation of α-Nrxn. Thus, expression of αDAG and Nxph1 together with alternative splicing in Nrxn1α may prevent or facilitate formation of distinct trans-synaptic Nrxn·Nlgn complexes, revealing an unanticipated way to contribute to the identity of synaptic subpopulations.

## Teaser

**Background:** Extracellular matrix dystroglycan has essential functions at the neuromuscular junction and at inhibitory synapses in the brain.

**Results:** Brain dystroglycan competes with neurexophilin-1 and neuroligins for binding to presynaptic α-neurexins.

**Conclusion:** Competition between α-neurexin ligands in combination with alternative splicing determines formation of important trans-synaptic complexes.

**Significance:** This is the first analysis of binding interference in α-neurexin multiplexes.

## Introduction

Neurexins (Nrxn)^2^ are transmembrane proteins that localize primarily to presynaptic terminals (1). Nrxn are essential for Ca^2+^-dependent transmission at excitatory and inhibitory synapses in the central and peripheral nervous system (2–6) and play additional roles in synapse formation and differentiation (7–12).

All three vertebrate Nrxn genes (*Nrxn1–3*) encode two major isoforms: extracellularly longer α-Nrxn and shorter β-Nrxn that are transcribed from independent promoters but share most exons (13). α-Nrxn proteins contain six LNS (laminin-neurexin-sex hormone binding globulin) domains with three interspersed EGF (epidermal growth factor-like) domains. β-Nrxn have a unique N-terminal stretch of 37 histidine-rich residues but are identical to α-Nrxn starting from LNS6 and ending in a cytosolic domain with PDZ binding motif that is required for trafficking (14). LNS domains are structurally characterized by a β-sheet sandwich (15–18), a core-fold similar to the concanavalin A family (19). LNS domains are thought to behave like glycan binding lectins (18), and LNS domains of Nrxn, laminin, and agrin have similar Ca^2+^ coordination sites (20). Nrxn LNS2 and LNS6 are distinguished by hydrophobic residues near the Ca^2+^-binding site, providing specific interfaces for binding partners (20, 21). Moreover, Nrxn contain up to six alternative splice sites in α-Nrxn (SS#1–6) and two in β-Nrxn (SS#4 and SS#5) (13, 22). Nrxn alternative splicing is physiologically relevant because it controls aspects of their function (23, 24) and binding to postsynaptic Nlgn (25, 26).

Splice insert-dependent formation of the Nrxn·Nlgn complex is intricate because Nlgn also has two splice sites. Nlgn1 contains SS_A and SS_B (26), Nlgn2 and Nlgn3 carry only SS_A (27), and Nlgn4 is not alternatively spliced (28). Co-crystal data exist for the interface between Nrxn LNS6/βLNS lacking insert in SS#4 (−SS#4) with Nlgn1 and Nlgn4 (15–17). Nlgn3 is predicted to form similar complexes (15–17), whereas Nlgn2 differs structurally with a G500Q change and may use another epitope (17, 29). Affinity purification of Nlgn with the extracellular domain of β-Nrxn originally suggested that only β-Nrxn(−SS#4) binds Nlgn1 (26). This apparent restriction fostered the idea of a splice code in Nrxn·Nlgn complexes (11, 25, 26, 30, 31). Subsequently, it was shown that all Nrxn, including α-Nrxn(+SS#4), are able to bind to Nlgn1(−B) or Nlgn2 due to displacement of the insert (20, 25, 32, 33).

In addition to Nlgn, other extracellular partners of Nrxn were identified, most notably neurexophilin (Nxph) (34–36), dystroglycan (DAG) (37), leucine-rich repeat proteins (LRRTM2) (38, 39), and cerebellin (40, 41). Remarkably, all of these ligands bind at only two Nrxn domains, LNS2 and LNS6/βLNS. Although Nxph binds LNS2 independently of alternative splicing (34), DAG and LRRTM require splice insert-free LNS domains (37, 42), and cerebellin binds directly to the insert in SS#4 (40, 41).

Unlike Nrxn variants that are expressed in most excitatory and inhibitory neurons (43), the α-Nrxn-specific ligand Nxph1 is restricted to inhibitory interneurons (36, 44), similar to αDAG, which also prefers subsets of inhibitory synapses where it may co-localize with Nlgn2 (45–48). Nxphs comprise a family of glycoproteins (Nxph1–4) that exhibit characteristics of secreted, preproprotein-derived molecules (35, 36), but the structural determinants of their interaction with LNS2 (34) are still unclear. DAG in turn is produced from an evolutionarily conserved single gene (49) and proteolytically cleaved into extracellular αDAG and transmembrane β-DAG that remain non-covalently attached (50). Although specificity of αDAG binding to matrix proteins such as laminin comes from glycosylation of distinct residues in the mucin-rich regions (51, 52), the glycan moiety required for association of Nrxn (37, 53) is undetermined.

Here, we present the distinct interaction sites of Nxph1 and αDAG at the LNS2 and study their cross-talk with ligands of the LNS6 domain of α-Nrxn. Surprisingly, we observed that binding of αDAG and Nxph1 is mutually exclusive and that association of αDAG at LNS2 prevents formation of the trans-synaptic complex with Nlgn at LNS6. These are important results because impairments in α-Nrxn·Nlgn complexes are linked to neurodevelopmental disorders (54–56), and there is symptomatic overlap with cognitive defects observed in DAG-associated muscular dystrophy syndromes (53, 57, 58).

## EXPERIMENTAL PROCEDURES

### Molecular Cloning and Expression Constructs

Previously described plasmids used include: full-length rat Nrxn1α, pCMVL2; Fc-tagged extracellular domains of Nrxn1α, pCMVIgN1α-1; Fc-tagged Nrxn1β, pCMVIgN1β-1, and pCMVIgN1β-3 (59); Fc-control (IgG) vector, pCMVIgNrxnSP; pCMVIgLNS6–1 without and pCMVIgLNS6–3 with insert in SS#4; mutations in LNS6–1 GT1201VA, L1280S/I1282S/N1284D, T1281A, D1183A; cassette pCMVIgLNS5-EGF3-LNS6; extracellular domains of rat Nlgn1 without insert B, pCMVNL1-B (20); full-length rat Nxph1, pCMVD2 (36); rat Nlgn2, pCMVNL2-1 (27); full-length rat DAG, pCMVDAG (60); full-length human LARGE (like-acetyl-glucosaminyl-transferase), pCMV6-XL4 LARGE (OriGene Technologies).

### Novel Plasmids Generated for This Study

#### 

##### Nrxn1α Constructs

single LNS domains (LNS1 to LNS6) and cassettes (LNS1-EGF1-LNS2, LNS3-EGF2-LNS4) were amplified from pCMVL2 and cloned into pCMVIgNrxnSP to add a C-terminal Fc tag. Site-directed mutagenesis on template Fc-LNS2 was done by QuickChange (Agilent Technologies, Santa Clara, CA) to introduce mutations C293S, S304A/S305A, Q316V, S327A, D329A, G347A, S348A, V358D, E356A, N359A, A366W, T395D, I401D, T403D, T403P/T404P/T405P, T404P, Q409A, Q409A/E410A/D411A, Y412A, Y412F, Y412S/M414S/G416D, Y412L/M414I/G416N, T413A, M414A, G416A, G416N, D418A, D418A/D419A, F420D, ΔS428–436P, L462R, and I472P. Mutation I1282R was based on Fc-LNS6. For C-terminally hemagglutinin (HA)-tagged LNS2 (LNS2-HA) and LNS6 (LNS6-HA), the HA sequence followed by a stop codon was introduced in Fc-LNS2 and Fc-LNS6. Soluble, non-tagged extracellular domains of Nrxn1α (amino acids ^31^LEF*X*_1315_EST^1346^) (+SS#4 and −SS#4) were created by inserting a stop codon in pCMVIgN1α-1.

##### Nxph1 Constructs

The mature domain of Nxph1 (amino acids MFGWGD*X_n_*PYFPSG) was cloned into Fc vector encoding for cleaved Nxph1, the protease Xa cutting site, followed by the Fc domain (pCMVIgNxph1*mat*). N-terminal and C-terminal domains of Nxph1 were obtained by deletion (Fc-Nxph1*mat*-NT, Fc-Nxph1*mat*-CT), leaving the epitope for anti-Nxph1 (loop antibody) intact. Site-directed mutagenesis was performed on pCMVIgNxph1*mat* to successively remove *N*-glycosylation sites (Fc-Nxph1*mat*-N156D, Fc-Nxph1*mat*-N156D-N162D, Fc-Nxph1*mat*-N146D/N156D/N162D, 3xND). Nxph1 without disulfide bonds were generated by mutating all 6 Cys (Fc-Nxph1mat-C210S, Fc-Nxph1mat-C210S/C218S (CS-a), Fc-Nxph1mat-C210S/C218S/C194S, Fc-Nxph1mat-C210S/C218S/C194S/C231S (CS-a-b), Fc-Nxph1mat-C210S/C218S/C194S/C231S/C239S, Fc-Nxph1mat-C210S/C218S/C194S/C231S/C239S/C256S (CS-a-b-c)).

##### αDAG Constructs

for Fc-tagged full-length αDAG the coding region (amino acids ^28^HWP*X*_623_TRG^651^) was amplified from pCMVDAG and inserted in Fc-vector (Fc-αDAG). Site-directed mutagenesis was done to deglycosylate αDAG at Asn-139, Thr-315, or Thr-317 and to delete either half or the complete mucin region (Fc-αDAG-N139D, Fc-αDAGmuc2 (Δmuc1), Fc-αDAGmuc1 (Δmuc2), Fc-αDAGΔmuc, Fc-αDAG-T315A/T317A). A soluble αDAG with a C-terminal HA tag was created by inserting an HA-stop sequence (αDAG-HA).

All enzymes for restriction sites, dephosphorylation, ligation, and appropriate buffers were purchased from New England Biolabs (Ipswich, MA). Custom-made primers were made by Sigma. PCR was carried out with iProof^TM^ high fidelity PCR (Bio-Rad), and DNA fragments were isolated using phenol-chloroform extraction or QiaEx (Qiagen, Hilden). All resulting intermediaries and final constructs were confirmed by DNA sequencing (GATC Biotech, Konstanz, Germany).

##### Biochemical Procedures

For expression of Fc-tagged recombinant proteins, constructs were transfected into HEK293, tsA-201, or N2a cells using calcium phosphate. Briefly, 28 μl of 8.4–17 μg of DNA in TE (10 mm Tris, pH 8, 1 mm EDTA) was premixed with 672 μl of 150 mm CaCl_2_ and 700 μl of phosphate buffer (274 mm NaCl, 12 mm glucose, 10 mm KCl, 1.4 mm Na_2_HPO4, 40 mm HEPES, pH 7.04), incubated at room temperature for 20 min, and added to 1.4 × 10^6^ HEK cells in growth medium (DMEM, 10% FCS, 5% penicillin/streptomycin). Medium was changed to FCS-free medium after 24 h, and recombinant proteins were harvested 72 h after transfection. Full-length Nrxn1α, Nlgn1, Nlgn2, and αDAG-HA were produced in COS7 cells using DEAE-dextran transfection. Briefly, 0.5 × 10^6^ cells in 10-cm dishes were washed twice with prewarmed (37 °C) 1× TBS, transfected with 3.3 ml of 1.65 ml of 2× TBS, 1.25 ml of H_2_O, 66 μl of DNA (0.1 μg/μl), and 330 μl of DEAE-dextran (5 mg/ml), and incubated for 30 min at 37 °C, 5% CO_2_. Medium was changed (DMEM, 10% FCS, 1% penicillin/streptomycin), 100 μm chloroquine was added, and cells were incubated for 3 h at 37 °C, 5% CO_2_. Medium was changed, and cells were incubated for 2 days before harvest. To produce deglycosylated Nxphs, tunicamycin (2 μg/liter) was added 24 h after transfection to FCS-free medium.

For co-sedimentation assays, secreted Fc-tagged proteins were bound to Protein A-conjugated Sepharose beads overnight, washed three times, and either analyzed directly or used in pulldown experiments essentially as described (20). In short, mouse brains were disrupted with a Polytron followed by Dounce homogenization in buffer H (100 mm NaCl, 5 mm CaCl_2_, 50 mm Tris, pH 7.5). Triton X-100 was added to a final concentration of 1% (w/v) for 3 h at 4 °C followed by centrifugation at 220,000 × *g* for 30 min. COS-7 or N2a cell lysates were obtained from scraped cells with 1% Triton X-100 in buffer H for 30 min at 4 °C and centrifugation (15,000 × *g*, 1 min). Aliquots of lysate were added to purified Fc fusion proteins in buffer H containing 0.1% Triton X-100 for binding at 4 °C overnight. After washing, bound proteins were analyzed by SDS/PAGE, Coomassie staining, and/or immunoblotting (Bio-Rad).

To obtain a pure Fc-Nxph1·LNS2-HA complex for mass spectrometry, we eluted free and LNS2-HA-bound Fc-Nxph1 from protein A beads with glycine buffer (50 mm glycine, pH 1.8) for 30 min, neutralized the eluate to pH 7 with 1 m Tris/HCl pH 8, separated the protein mixture on a Superdex 200 gel filtration column XK 16, and collected the fraction containing only Fc-Nxph1·LNS2-HA complex using an Äkta^TM^ prime system (GE Healthcare). The protein solution was concentrated to 500 μl by Amicon ultracel filters (3K) and dialyzed against 10 mm ammonium bicarbohydrate in Slide-A-Lyzer^TM^ chambers (Thermo Scientific) before mass spectrometry analysis.

##### Surface Plasmon Resonance (SPR) Analysis

Fc-Nxph1·LNS2-HA eluted from protein A beads as described above was bound covalently on a CMD 200m chip using 5 mm sodium acetate, pH 5, and 1-ethyl-3-(3-dimethyl-aminopropyl)carbodiimide, *N*-hydroxysuccinimide (EDC-NHS) following the manufacturer's protocol (Reichert Technologies, New York). Binding occurred randomly in a flow of 50 μl/min, and stochastically either Nxph1, LNS2, or both proteins were irreversibly immobilized on the chip. 50 mm Tris, pH 7.4, served as the running buffer, and several solutions were tested to release either Nxph or LNS2 from their complexes. Complexes that were covalently linked to the chip via both proteins remained inert to elution buffers used, leading to a systematic underestimation of the eluted fraction under all conditions that did not bias our relative comparison between WT and point-mutated complexes. Measurements were done with a two-channel Reichert SR7000DC SPR System (Reichert Technologies).

##### Mass Spectrometry

Nxph1 samples were digested in ammonium bicarbonate buffer (10 mm) overnight with trypsin, chymotrypsin, or a 1:1 mixture of trypsin and chymotrypsin at 37 °C or with thermolysin at 65 °C. Digest mixtures were dried, redissolved in water, and dried again. For separation of *N*-glycopeptides, ZIC-HILIC ProteaTips were used as described previously (61). Products were analyzed by nanoESI quadrupole time-of-flight mass spectrometer (Micromass, Manchester, UK) equipped with a Z-spray source in positive ion mode. Spectra were acquired at source temperature of 80 °C, a desolvation gas (N_2_) flow rate of 75 liters/h, a capillary voltage of 1.1 kV, and a cone voltage of 30–40 V. For low energy collision-induced dissociation experiments, the (glyco)peptide precursor ions were selected in the quadrupole analyzer and fragmented in the collision cell using a collision gas (Ar) pressure of 3.0 × 10^−3^ Pa and collision energies of 20–40 eV (Elab). The (glyco)peptide structures were deduced from the resulting fragment ion spectra (Table 1).

##### Immunoelectron Microscopy

Neocortical brain tissue from wild-type mice was embedded in Lowicryl HM20 (Polysciences, Eppelheim, Germany) using freeze substitution in methanol. Anesthetized mice were transcardially perfused with 0.1% glutaraldehyde (Roth, Karlsruhe, Germany) and 4% paraformaldehyde (Merck) in 0.1 m phosphate buffer at 37 °C and postfixed for 2 h. 300-μm-thick vibratome slices were infiltrated for cryoprotection with 5% sucrose in 0.1 m phosphate buffer followed by 10, 20, and 30% glycerol in 0.1 m phosphate buffer for 2 h each. Blocks were cut and plunged into 4% uranylacetate in 99.5% methanol precooled to −90 °C in a Leica AFS2 for 12 h and additional 24 h at −45 °C (slope 5 °C/h). After 3 washing steps in methanol (30 min at −45 °C) infiltration followed for 2 h with 50% Lowicryl in methanol, 67% Lowicryl in methanol, and pure Lowicryl for an additional 16 h. Polymerization with UV light proceeded for 24 h at −45 °C followed by 24 h at 0 °C (slope 4 °C/h) and finally 24 h at room temperature (slope 0.9 °C/h). Post-embedding immunogold labeling on Lowicryl sections started by blocking on 2% HSA, 0.05 m TBS droplets followed by incubation with primary antibodies against Nxph1 (1:50), Nrxn (1:50), Nlgn2 (1:50), Nlgn1 (1:50), and pan-synapsin (1:50) overnight at 4 °C and 10 nm-gold antibody for 2 h at room temperature. Labeled sections were washed in TBS and contrasted with saturated uranylacetate. Samples were investigated with a transmission electron microscope (Libra 120, Zeiss, Germany) at 80 kV, and images were taken with a 2048 × 2048 CCD camera (Tröndle, Moorenweis, Germany).

##### Antibodies

Rabbit anti-Nrxn (A473) (1) and anti-synapsin (E028) (62), mouse anti-Nlgn1 (4C12, Synaptic Systems, Göttingen, Germany), anti-Nlgn2 (Synaptic Systems), anti-αDAG (VIA4–1, IIH6C4, Upstate/Millipore, Billerica, MA), anti-HA (12CA5, Roche Applied Science, HA.11 clone 16B12, Covance, München, Germany), and a new affinity-purified rabbit anti-Nxph1 against peptide AQQTVIDAKDSKSC in the variable linker between conserved domains (Eurogentec, Liège, Belgium) were used for immunolabeling.

##### Structural Modeling

SwissProt entries NRX1A_RAT (Q63372-7), NXPH1_RAT (Q63366), and DAG1_Mouse (Q62165) were used to generate models (Figs. 3*A*, 4*E*, 6, 7*C*, and 8*A*). Gas6·Axl (PDB code 2C5D) served as template for the LNS2·Nxph1 complex, in which LNS2 (PDB code 2H0B) replaces Gas6. Axl residues Thr-204 to Lys-208 served as the backbone for the β-β sheet interaction coordinates, and the Nxph1 sequence was passed in single amino acids steps through this backbone structure to generate all possible LNS2·Nxph1 complexes. Each peptide complex was scored by calculating stability with FoldX. The complete C-terminal domain of Nxph1 was then homology-modeled using PAF (PDB code 2KCN) that contains an identical cysteine pattern, whereas the N-terminal domain was modeled by threading with PHYRE2. Both domains were manually connected and glycosylated using GLYCAM carbohydrate builder. An alternative model of the Nxph1 C-terminal domain was generated using coordinates of snake neurotoxin (PDB code 1NTN). For pictograms of αDAG, the mucin region was modeled using PHYRE2 (80) and a distorted structure as template (PDB code 4A54). The C-terminal domain as described (63) was modeled using the N-terminal domain structure (PDB code 1U2C), and parts were manually assembled and glycosylated with GLYCAM. For complex presentation, αDAG was manually docked to the Nrxn1α structure (PDB code 3R05), whereas Nlgn1 and Nlgn2 dimers were placed according to co-crystals (PDB code 3B3Q). All structures were rendered and visualized using PyMOL.

## RESULTS

### 

#### 

##### αDAG, Nxph1, and α-Nrxn Are Expressed at Inhibitory Synapses

Although localization of αDAG at GABAergic terminals could be demonstrated by immunocytochemistry (45, 48), the hypothesized presence of Nxph1 relied on indirect evidence from *in situ* hybridization data (36) and functional deficits observed in electrophysiological recordings from knock-out (KO) neurons (64).

**FIGURE 1.**
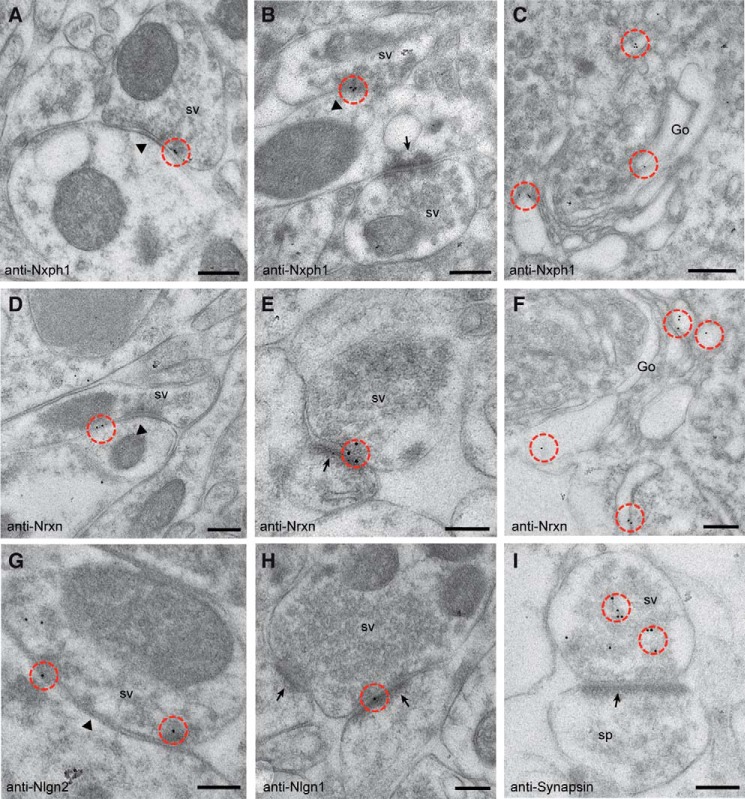
**Ultrastructural localization of endogenous Nxph1.** Immunoelectron microscopy of Lowicryl-embedded neocortical tissue from murine brain was used to determine the exact localization of Nxph1 (*A-C*) and its cognate receptor Nrxn (*D–F*). *sv*, synaptic vesicle. Post-embedding with 10-nm gold-labeled secondary antibodies reveal Nxph1 only at symmetric, type 2 terminals (*A* and *B*, *arrowheads*), whereas asymmetric, type 1 contacts (*B*, *arrows*) are devoid of gold particles (*circled in red in all panels*). Nrxn is seen at both type 2 (*D*, *arrowhead*) and type 1 (*E*, *arrow*) synapses, representing inhibitory and excitatory terminals, respectively. *C* and *F*, labeling of Nxph1 and Nrxn in Golgi cisternae (*Go*) demonstrate their passage through the secretory pathway. *G–I*, control experiments showing the predicted differential distribution of the trans-synaptic Nrxn ligand Nlgn2 at symmetric (*G*) and Nlgn1 at asymmetric (*H*) synapses. *I*, a different labeling pattern is observed with anti-synapsin antibodies, confirming its association with synaptic vesicles in the presynaptic terminal of a type 1 spinous contact (*sp*). *Scale bars*, 200 nm, except in *C* and *F*, 300 nm.

Here, we used immunogold electron microscopy to probe the ultrastructural localization of endogenous Nxph1 in cortical tissue of adult mice. To distinguish between actual localization and residual background from a polyclonal antiserum raised against the loop region (see “Experimental Procedures”), we compared labeling patterns in wild-type samples with KO and performed control labeling without first antibody using Lowicryl-embedded brain tissue. Although negative controls showed essentially no labeling (data not shown), Nxph1 normally localizes specifically to membranes of symmetric, inhibitory synapses in the neocortex (Fig. 1*A*). Asymmetric contacts, corresponding to excitatory synapses, were not labeled (*arrow* in Fig. 1*B*), whereas Nxph1 concentrated at the synaptic cleft of symmetric profiles (*arrowhead* in Fig. 1*B*) or in compartments of the secretory pathway such as rough endoplasmatic reticulum or Golgi cisternae (Fig. 1*C*), expected for a neuropeptide-like protein (35). To validate these observations, we also tested the ultrastructural distribution of its cognate receptor Nrxn and the trans-synaptic interaction partners of Nrxn, Nlgn1, and Nlgn2 in the same samples. We observed Nrxn at the synaptic cleft of both symmetric (Fig. 1*D*) and asymmetric (Fig. 1*E*) contacts in addition to localization in the secretory pathway (Fig. 1*F*), consistent with their widespread expression and function in inhibitory and excitatory synapses (4, 43). Demonstrating the reliability of our protocol, we could confirm the subtype-specific distribution of Nlgn2 at inhibitory (Fig. 1*G*) and Nlgn1 (Fig. 1*H*) at excitatory synapses as reported (47, 65). To finally brace against artifacts from our post-embedding procedure that may bias localization toward plasma membranes, we applied a pan-synapsin antibody but observed the expected different pattern over synaptic vesicles in presynaptic profiles (Fig. 1*I*). We conclude from our current and published results that Nxph1 is actually present at inhibitory synapses along with α-Nrxn, Nlgn2, and αDAG, providing a rationale for biochemical investigations of α-Nrxn/Nxph1-based multiplexes that might play a specific role at the GABAergic synaptic subpopulation.

##### αDAG Binding to Nrxn

**FIGURE 2.**
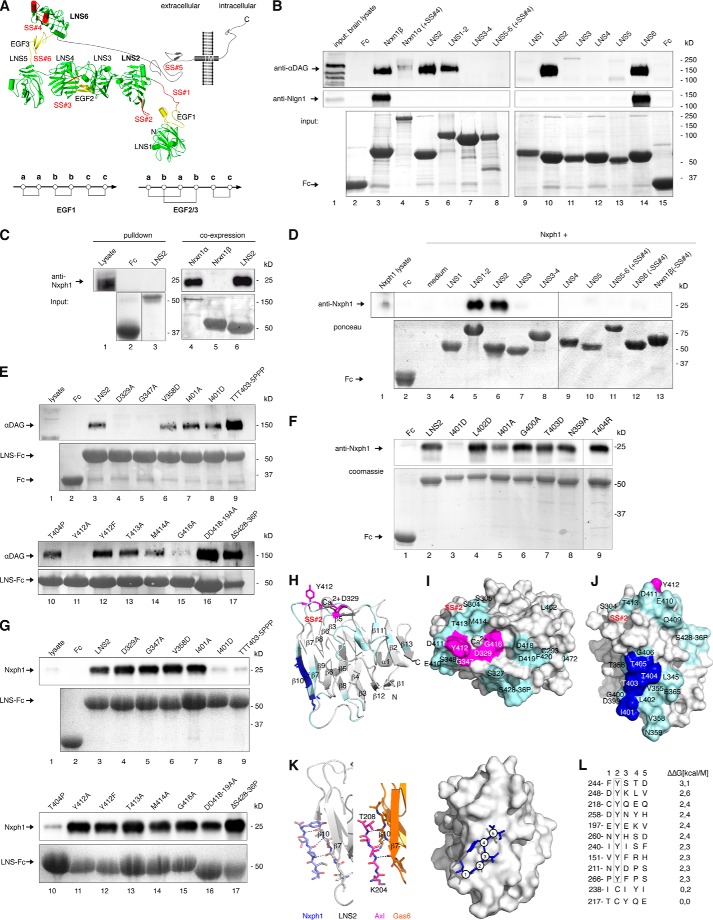
**αDAG and Nxph1 bind the same αNrxn LNS domain but different epitopes.** *A*, modeled Nrxn1α structure in u-form conformation, modified from (33). Flexibel position of LNS1 is extended by an unusual *aabbcc* disulfide pattern of the EGF1 domain (see the *inset below*) but likely constrained to an area proximal to the presynaptic membrane. Up to six alternative splice sites (SS#1–6) accept inserts (*red*). The stalk region between LNS6 and transmembrane region (*TM*) is visualized as rod-like due to massive *O-*glycosylation (119). *B*, binding of αDAG (immunoblots, *upper panel*) and Nlgn1 (*middle panel*) from mouse brain was tested by pull-down with Fc-tagged extracellular domain of Nrxn1α carrying an insert in SS#4 (*lane 4*). Interactions of the two ligands to all six LNS domains (*lanes 5* and*9–14*), three LNS-EGF-LNS cassettes (*lanes 6–8*), and to Nrxn1β (*lane 3*) were tested, and amounts of purified Fc-tagged Nrxn domains were visualized by Coomassie staining (*lower panel*). Fc protein was used as negative control (*lanes 2* and *15*). *C*, transfection of HEK293 cells with full-length Nxph1 produces mature protein of 25kD (*lane 1*) that does not bind to Fc-tagged LNS2 in pulldown assays (*lane 3*, immunoblot, *upper panel*; Coomassie, *lower panel*). Co-expression of Nxph1 with Fc-tagged Nrxns allows complex formation and purification of Nxph1·Nrxn1α-Fc (*lane 4*) and Nxph1·LNS2-Fc (*lane 6*) from culture medium, whereas Nrxn1β does not bind to Nxph1 (*lane 5*). *D*, co-expression of Nxph1 with Fc-tagged Nrxn1α domains containing LNS2 yields bound Nxph1 (*lanes 5* and *6*), whereas other domains do not bind (*lanes 4* and *7–13*). Co-expression of Nxph1 with Fc was used as negative control (*lane 2*); in cultures transfected with Nxph1 alone, the protein is hardly detectable in lysate (*lane 1*) and medium (*lane 3*). *E–G*, site-directed mutagenesis to identify LNS2 residues required for binding of αDAG or Nxph1. *E*, binding of αDAG from mouse brain lysate (*lane 1*) to Fc-tagged wild-type (*lane 3*, immunoblot (*upper panel*); protein staining (*lower panel*)) or mutated (*lanes 4–17*) LNS2 domains was tested by pulldown assay. Individual mutations are described in “Results”. *F*, binding assay after co-expression of Nxph1 with WT (*lane 2*) or mutated (*lanes 3–9*) LNS2-Fc in cell culture. *G*, mutations preventing αDAG binding (*E*) still bind to Nxph1 (*lanes 4–5*, *11*, and *16*, *upper panel*). In the reverse, mutations of residues Ile-401 (*lane 8*, *upper panel*), Thr-403, Thr-404, or Thr-405 (*lanes 9* and *10*, *upper panel*) block Nxph1 binding. Co-expression of Nxph1 with Fc was negative (*F*, *lane 1*; *G*, *lane 2*). *H*, ribbon structure of LNS2 with αDAG binding epitope (*magenta*) near the calcium co-coordination site and epitope for Nxph1 (*blue*) at β10 strand. The approximate position of SS#2 in loop β8-β9 is in *red. I*, surface view of αDAG epitope (*magenta*). *J*, binding epitope for Nxph1 (*blue*); non-involved residues (*cyan* in *H–J*). *K*, Nxph1 site at β10 of LNS2 compares to Axl binding to LNS1 of Gas6 (PDB code 2C5D). Homology of LNS domains from Nrxn1α and Gas6 (*orange*, *middle*) allowed modeling of LNS2·Nxph1 peptide complexes (*left* and *right*). *L*, all possible 150 complexes were scored by the difference in free binding energy (ΔΔG, *right*) normalized to the best binding peptide. Peptides having a Tyr at position 2 are sterically hindered, and peptides with a Cys at this position gave the best results (ΔΔ*G* < 0.5 kcal/Mol).

Binding of brain αDAG to α-Nrxn was reported (37), but its structural determinants and consequences for other interaction partners of Nrxn remained open. An obstacle had been the lack of information on α-Nrxn conformation; however, recent crystal data of extracellular sequences (33, 66, 67) allowed us to study the effects of αDAG binding based on structural predictions. The structure of α-Nrxn consists of six LNS domains (Fig. 2*A*, *green*) intercepted by three EGF-like domains (Fig. 2*A*, *yellow*) that assemble into a rigid core of LNS2-to-LNS5 (33, 66). EGF2 and EGF3 show a typical *ababcc* cysteine knot pattern that tightly joins their adjacent LNS domains, whereas we determined here an *aabbcc* connectivity of EGF1 by mass spectrometry (Fig. 2*A*, LDE*X_n_*GVC, *m*/*z*_exp_ 1173.81; *m*/*z*_calc_ 1173.44) that can open a gap between LNS1 and LNS2 by more than 11 Å. Our observation explains the highly variable linkage of LNS1 (66, 68) and makes LNS2 accessible. The u-form conformation of α-Nrxn (Fig. 2*A*) opens the possibility that binding partners of the back-folded LNS2 domain interfere with ligands at the LNS6 domain. To address this important possibility, we first determined the exact binding epitopes of αDAG and Nxph1 using a combination of co-precipitation assays and site-directed mutagenesis as previously established (20).

Building on the sole previous study on αDAG-Nrxn interaction (37), we confirmed the binding of DAG to Nrxn1α and Nrxn1β and then tested all LNS domains individually (Fig. 2*B*). We found that αDAG from mouse brain interacts with both LNS2 and LNS6 (Fig. 2*B*, *upper panel*). This is an interesting result because only these domains, but none of the other four LNS, were shown to mediate all ligand binding (69), emphasizing the need to explore potentially competing complexes. Testing pulldown of Nlgn1 in the same experiments validated the known binding site at LNS6 (Fig. 2*B*, *lane 14*, *middle panel*) and Nrxn1β (*lane 3*) (15–17, 20, 70). In line with earlier studies which discovered that interaction of Nlgn1 and Nrxn depends on alternative splicing (25, 26, 71), Nlgn1 could not be pulled-down from brain lysates by full-length extracellular Nrxn1α(+SS#4) (Fig. 2*B*, *lane 4*, *middle panel*) or by LNS5-EGF3-LNS6 cassette with insert (Fig. 2*B*, *lane 8*, *middle panel*). αDAG also prefers splice insert-free LNS domains (37) and thus binds to LNS2, LNS6, and Nrxn1β without insert in SS#2 or SS#4 in our co-sedimentation assay (Fig. 2*B*, *lanes 3*, *5–6*, *10*, and *14*, *upper panel*). Because αDAG is able to interact with two LNS domains, it can interact with Nrxn1α(+SS#4, −SS#2) that has a blocked LNS6 but an insert-free LNS2 domain (*lane 4*). This is an interesting aspect as Nrxn in adult brains mostly contain (+SS#4) mRNA variants (22, 72), suggesting that alternative splicing in Nrxn may affect several ligands simultaneously. Although some knowledge on competitive interaction of Nlgns and LRRTM with Nrxn is available (42), it is unclear if αDAG also competes for the same epitope at LNS6 and how its binding to LNS2 is affected by Nxph (34).

To determine the site of Nxph1 binding, we had to develop a modified binding assay because normal pull-down failed (Fig. 2*C*). Instead, we co-expressed recombinant Nxph1 with Fc-tagged Nrxn1 constructs in HEK293 cells, precipitated the pre-formed Nxph1·Nrxn-IgGFc complexes secreted into culture media with protein A beads, and tested binding by immunoblotting (see “Experimental Procedures” for details). This approach also allowed the reverse experiment with mutated Nxph1 residues, which were virtually impossible to accomplish by adenovirus-mediated transfer used previously to generate sufficient amounts of Nxph (34). Using the co-expression assay, we confirmed the binding of Nxph1 to isolated LNS2 (Fig. 2*D*, *lane 6*) and to the LNS1-EGF1-LNS2 cassette (*lane 5*), whereas other Nrxn1α domains (*lanes 4*, and *7–12*) or Nrxn1β (*lane 13*) do not bind.

##### Interaction Sites of αDAG and Nxph1 on LNS2

To study the characteristics of αDAG and Nxph1 binding epitopes on LNS2, wild-type and mutated Fc-tagged LNS2 domains were immobilized on beads and tested for their ability to precipitate endogenous αDAG from brain lysate (Fig. 2*E*). As predicted from its calcium dependence (37), αDAG binding is diminished by alanine mutations of calcium-coordinating residues Asp-329 (*lane 4*, *upper panel*) and Gly-416 (*lane 15*, *upper panel*). More unexpectedly, nearby residues Gly-347 (*lane 5*, *upper panel*) and Tyr-412 (*lane 11*, *upper panel*) also influence binding. Because the hydroxyl group of Tyr-412 does not participate in αDAG association as indicated by mutation Y412F (*lane 12*, *upper panel*), these results suggest that hydrophobicity of these Nrxn residues is required for αDAG binding, in contrast to a basic epitope in laminin interacting with αDAG (73, 74). Although glycosylation of αDAG is essential for Nrxn binding (37, 53), deletion of the LNS2 loop β11-β12 (residues 428–438), recently proposed to contain a carbohydrate binding site (33), demonstrates that it is not involved in αDAG binding (Fig. 2*E*, *lane 17*, *upper panel*). Similarly, many additional residues were tested by site-directed mutagenesis but do not influence αDAG association (*lanes 6–10*, *13*, *14*, and *16*, *upper panel*). We mapped these data to the surface of the LNS2 structure, resulting in delineation of the αDAG epitope (*magenta* in Fig. 2, *H–J*).

Similar to αDAG, little is known about the Nxph1 binding epitope; therefore, we tested the entire surface of LNS2 to determine residues required for the Nxph1·LNS2 interface. For example, we deleted distinct loops like residues 428–436 (Fig. 2*G*, *lane 17*) and changed hydrophobic to charged residues at strategic positions. We observed that the interface requires hydrophobicity of residue Ile-401 (Fig. 2*F*, *lane 3*, *upper panel*) but not its entire side chain (*lane 8*). Nearby residues, including Leu-402 (*lane 4*) and threonines 403–405 (*lanes 7* and *9*), showed no change in Nxph1 binding when mutated to charged side chains. The only free cysteine, Cys-293 (Fig. 2*I*), and numerous amino acids showed no effect (Figs. 2, *F*, *lanes 6* and *8*, *G*, *lanes 4–6* and *11–16*, *upper panel* and *cyan* in *I-J*). Because Ile-401 is part of the β10 strand in LNS2, we asked if Nxph1 engages in side chain-independent β-β interactions. We mutated the central residues of β10 to prolines and observed loss of Nxph1 complex formation (Fig. 2*G*, *lanes 9–10*), whereas binding to αDAG persisted (Fig. 2*E*, *lanes 9–10*). These data explain why binding between Nxph1 and α-Nrxn occurs calcium- and splice site-independently (34); the binding epitope at LNS2 (*blue* in Fig. 2, *H* and *J*) is distant to these positions and also non-overlapping with the αDAG binding site (*magenta* in Fig. 2, *H–J*).

In a first attempt to assemble the Nxph1·LNS2 complex by bioinformatics, we identified the crystal structure of Gas6 LNS1·Axl as a structural template. The interaction of β-strand residues 204–208 of Axl with the correspondent β10 of Gas6 LNS1 (Fig. 2*K*, *middle panel*) is not their only contact interface (75) but best suits our purpose of modeling a Nxph1·LNS2 peptide complex (*left* and *right panel*s). We generated models of all 150 possible LNS2·Nxph1 peptide combinations and calculated the relative change in free binding energy (ΔΔ*G*, Fig. 2*L*, *right*). These results show that any sequence of 5 residues of Nxph1 will bind to β10 of LNS2 with the only exception that a tyrosine is not allowed at position 2. This restriction only limits the number of potential complexes to 140, indicating that a mutagenesis study of single positions in Nxph1 has to await more structural information.

##### Nxph1 Prevents Simultaneous Binding of αDAG at LNS2

**FIGURE 3.**
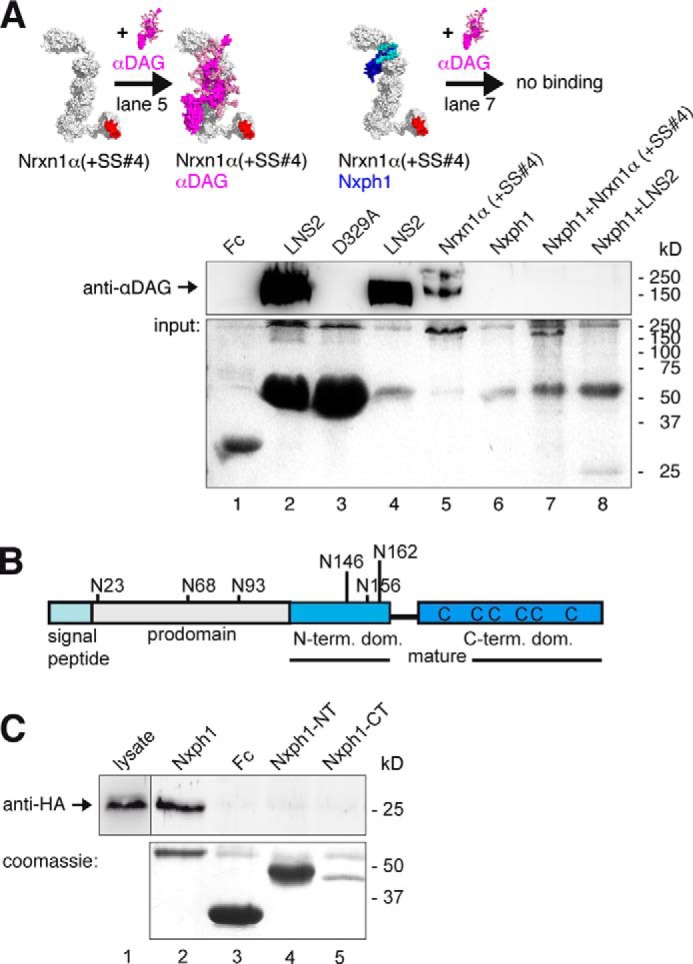
**Nxph1 in complex with α-Nrxn prevents binding of αDAG to LNS2.** *A*, pictograms of interactions probed (color-coded as labeled; splice inserts are in *red*) and representative immunoblot. Endogenous αDAG from N2a cells binds to Fc-tagged extracellular domain of Nrxn1α (*lane 5*) and to isolated Fc-LNS2 (*lanes* 2 and *4*). Preformed complexes of Fc-tagged Nxph1·Nrxn1α (*lane 7*) or Nxph1·LNS2-HA (*lane 8*) prevent αDAG binding. Protein staining after pull-down (*lower panel*) shows that low amounts of LNS2-Fc (∼55 kDa) are sufficient to bind αDAG (*lane 4*), but LNS2-HA (25 kDa) in complex with Nxph1-Fc (∼55 kDa) does not (*lane 8*). Comparable amounts of Nrxn1α-Fc (∼220 kDa, *lane 5*) and Nrxn1α (∼165 kDa, *lane 7*) were used. Isolated Nxph1-Fc does not bind to αDAG (*lane 6*). LNS2 and glycosylated mature Nxph1 are both ∼25 kDa (*lane 8* and Fig. 2*B*). *B*, preproprotein with signal peptide (*cyan*) and prodomain (*gray*) is cleaved to generate mature Nxph1, consisting of an *N*-glycosylated N-terminal (*light blue*) and a cysteine-rich C-terminal (*dark blue*) domain. *C*, binding of Fc-tagged Nxph1 to HA-tagged LNS2 by co-expression in COS7 cells (*lane 1*) requires complete mature protein (*lane 2*), as isolated N- (*lane 4*) or C-terminal domains (*lane 5*) are not sufficient. The Fc-tagged glycosylated N-terminal domain has a similar size as the non-glycosylated C-terminal domain. The size of molecules in *A* are to scale, and the modeled complexes αDAG·αNrxn and Nxph1·αNrxn were generated using two criteria, (i) coverage of hot spots and (ii) maximal surface area buried (see “Experimental Procedures”). Note that in addition to complexes shown, other conformations are not excluded.

Our identification of separate binding epitopes for αDAG and Nxph1 suggested that simultaneous binding of both LNS2 ligands should be possible. We obtained complexes of Fc-tagged Nxph1 with a soluble extracellular domain of Nrxn1α(+SS#4) or with LNS2-HA by co-expression that were purified and used to pull down αDAG from neuron-like N2a cells, a rich source of endogenous αDAG. Surprisingly, the Nxph1·Nrxn1α complexes could not interact with αDAG, and no triple complex was formed (Fig. 3*A*, *lanes 7* and *8*), whereas control pull-down with Nxph1-free Fc-Nrxn1α(+SS#4) (Fig. 3*A*, *lane 5*) or Fc-LNS2 (*lane 4*) reliably bound αDAG. These results indicate that the presence of Nxph1 may sterically constrain αDAG binding to α-Nrxn, prompting us to examine key aspects of the Nxph1 structure in α-Nrxn binding.

Based on sequence analysis (35, 36), Nxph1 was identified as a preproprotein with putatively secreted mature protein consisting of glycosylated *N*-terminal and cysteine-rich C-terminal domains (Fig. 3*B*). To determine the contribution of Nxph1 domains to complex formation with α-Nrxn, we probed if N-terminal or C-terminal sequences are involved and observed that both are required (Fig. 3*C*). In addition, we found that secretion of the C-terminal domain is reduced, possibly pointing to a role of the six cysteines in fold stabilization.

**FIGURE 4.**
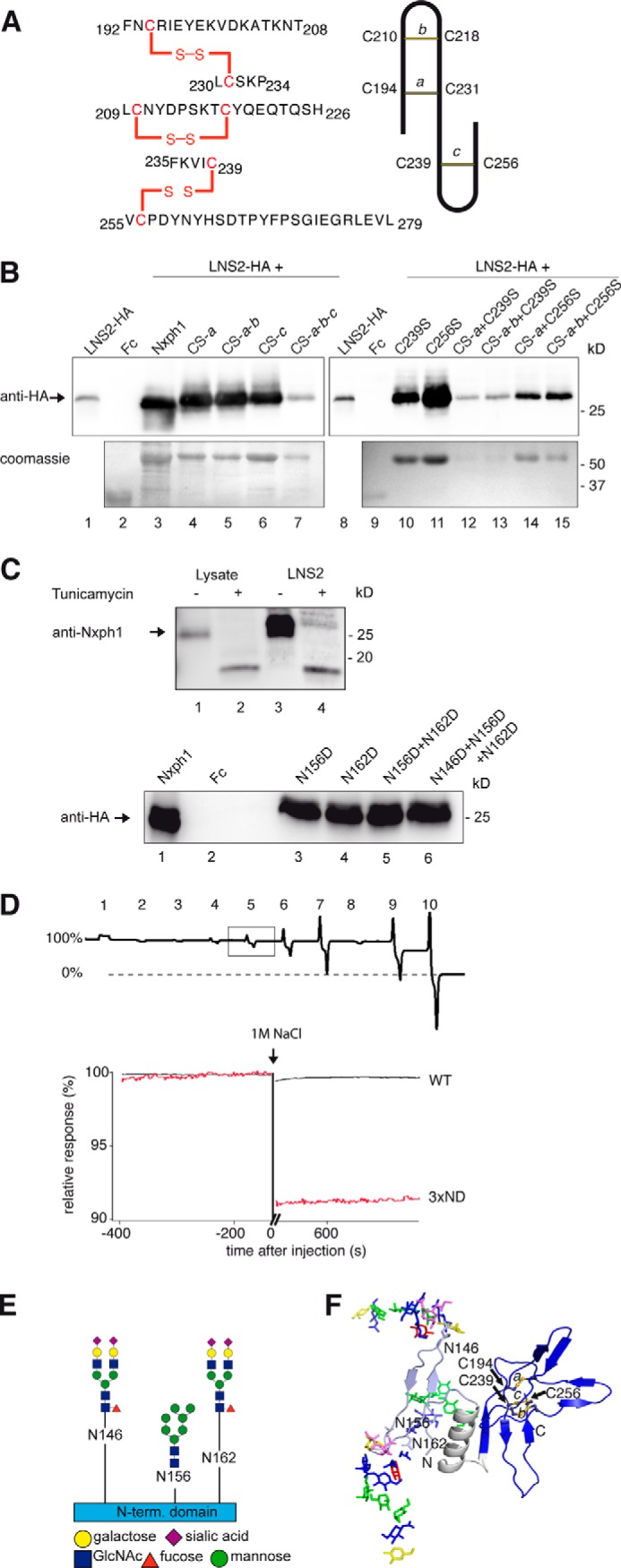
**Structural determinants of Nxph1·α-Nrxn complex formation.** *A*, nanoESI quadrupole time-of-flight mass spectrometry of recombinant Nxph1 protein (*left panel*) reveals a *abbacc* cysteine connectivity with three bridges (scheme, *right panel*). *B*, successive Cys-to-Ser mutations (*lanes 4–7*), analysis of single mutations (*lanes 10–11*), and their combinations (*lanes 12–15*) identify Cys-239 as most sensitive for Nxph1 binding to LNS2 (*lanes 12* and *13*). C239S reduces secretion of Nxph1 when combined with cysteine bridge *a* (*lanes 12–13*); similar combinations with C256S have no effect (*lanes 14–15*). *C*, *N*-glycosylation of Nxph1 is not required for complex formation with LNS2. Binding of recombinant mature Nxph1 to Fc-tagged LNS2 co-expressed in COS7 cells without (*lane 3*) and with (*lane 4*) the addition of tunicamycin (*upper panel*). Successive Asn-to-Asp mutations of all *N*-glycosylation sites (Asn-146, Asn-156, and Asn-162) did not prevent complex formation (*lanes 3–6*, *lower panel*). *D*, *N*-glycosylation stabilizes the Nxph1·LNS2 complex. In a reversed surface plasmon resonance experiment, purified Nxph1-Fc·LNS2-HA complex covalently linked to CMD chip was tested to dissolve by serial injection of 100 mm NaOAc pH4 (*1*), 50 mm Tris, pH 8.9 (*2*), 250 mm NaCl (*3*), 500 mm NaCl (*4*), 1 m NaCl (*5*), 2 m NaCl (*6*), 4 m NaCl (*7*), 5 mm EDTA (*8*), 6 m urea (*9*), and 7 m guanidinium chloride (*10*). The wild-type (*WT*) complex was not affected by most conditions (the base line not changed, 100% binding) but can be disassembled by denaturing agents (*9* and *10*), serving as the reference for releasable protein (0% binding). In contrast to WT (*square*, *upper panel; black trace*, *lower panel*), deglycosylation by the triple Asn to Asp mutation (*3*× *ND*) released 9% of the complex (*red trace*, *lower panel*) after injection of 1 m NaCl. *E*, glycosylation pattern of the Nxph1 N-terminal domain as determined by mass spectrometry (for details, see Table 1). Residues Asn-146 and Asn-162 were bound to complex type glycans containing fucose and sialic acids, and Asn-156 was linked to high mannose glycans. *F*, molecular model structure of Nxph1 with glycans attached.

The highly conserved cysteines in the C-terminal domain should help to classify its structural fold (76). However, bioinformatic prediction programs like Raptor (77), I-Tasser (78), Rossetta (79), or Phyre (80) failed to predict the cysteine connectivity or the fold. We, therefore, purified recombinant Fc-tagged mature Nxph1 in complex with LNS2-HA and analyzed the structure by mass spectrometry methods (81). Our co-expression system produced two secreted protein fractions, free Nxph1_Fc and Nxph1_Fc, bound to LNS2-HA in a ratio of ∼2:1 that we separated by gel filtration. Surprisingly, mass spectrometry revealed disulfide bonds in an *abbacc* pattern (Fig. 4*A*, *left* and *right panels*) and not a more frequent cysteine knot, which is present, for example, in Nrxn1α EGF2 and EGF3 (Fig. 2*A*) (33, 66). This connectivity was the same in free and LNS2-bound Nxph1. Because this *abbacc* pattern is rare but might be fold-stabilizing (82, 83), we successively opened all bridges by cysteine to serine mutations (*CS*, *a* to *a-b-c*) and found that any two bridges can be opened at the same time without an effect (Fig. 4*B*, *lanes 4–6*, *10*, *11*, *14*, and *15*, *upper panel*). However, asymmetric triple mutations containing C239S strongly reduce Nxph1 secretion (*lanes 12* and *13*, *lower panel*). Similarly, opening of all three cysteine bridges reduced both binding capabilities (*lane 7*, *upper panel*) and secretion (*lower panel*), which is likely explained by a destabilized fold (84, 85). The strong effect by opening the third bridge (*lanes 7* and *10–15*) highlights Cys-239 as a key residue in fold stabilization. Although these results demonstrate that Nxph1 contains a rare fold with unusual cysteine pattern required for its own secretion bound to α-Nrxn, they do not solve the question of why additional binding of αDAG to preformed Nxph1·α-Nrxn is blocked.

Because the N-terminal domain of Nxph1 is also involved in complex formation (Fig. 3*C*, *lane 5*), we evaluated the contribution of *N*-glycosylation, its distinctive feature. *N*-Glycosylation is not a prerequisite for complex formation as shown by tunicamycin treatment (Fig. 4*C*, *upper panel*) and point mutations of all three relevant residues including triple mutation N146D/N156D/N162D (*lower panel*). Although this observation is consistent with earlier data (36), we now found in SPR experiments with preloaded Fc-Nxph1·LNS2-HA that the glycosylation is critical for complex stability (Fig. 4*D*); wild-type complex bound to SPR chips resisted stringent elution (*lanes 1–8*, *upper panel*), and only near-denaturing conditions (6 m urea, *lane 9*; 7 m guanidinium chloride, *lane 10*) dissolved the complex. However, the complex with non-glycosylated triple Asn to Asp mutations (3xND) already started to fall apart at 1 m NaCl (Fig. 4*D*, *ND*, *lower panel*), pointing to an unexpected role of Nxph1 glycans in strengthening the interaction with α-Nrxn. We, therefore, analyzed the glycosylation pattern by mass spectrometry and observed two sites occupied by complex type glycans and one by high mannose-type oligosaccharides (Fig. 4*E* and Table 1). Although complex type glycans with terminal sialic acids and core fucose as seen here on Asn-146 and Asn-162 of Nxph1 are unusual for such proteins (86), we observed the same *N*-glycans on its cognate receptor Nrxn1α (Table 1). The glycans identified add ∼4 kDa to the N-terminal domain, leading to similar molecular weights for both domains (Fig. 3*C*). To visualize the complete, glycosylated mature Nxph1, we generated a model structure with glycans (Fig. 4*F*). The *N*-glycosylated N-terminal domain is shown as a single β-turn (Fig. 4*F*, β1/β2, *light blue ribbon*) that is flexibly linked (*gray helical linker* and antibody epitope) to the C-terminal domain. Because only NMR data of the antifungal protein PAF (83) described an *abbacc* cysteine fold, we used these coordinates to model the C-terminal domain, which constitutes a three-leafed seven-stranded β-fold (β3-β9, *dark blue ribbon*) stabilized by three cysteines (*yellow sticks*). In contrast to PAF, we have determined constant connectivity, but cysteine isomerization can explain the stabilizing effect of asymmetric triple-mutation CS-*a*+C256S (Fig. 4*B*, *lane 14*), whereas CS-*a*+C239S appears unstable (*lane 12*). Assuming the same cysteine cluster as in the protein PAF, the free Cys-239 in CS-*a*C256S could form an alternative cysteine bond to Cys-194 of *a*, whereas vice versa a free C256 in CS-*a*+C239S was not in reach of *a*. From this model it is likely that the C-terminal domain will bind to β10 of LNS2 (Fig. 2*J*). In addition, the high mannose-type glycan on Asn-156 is likely to be buried in the interface with α-Nrxn to protect Nxph1 from ubiquitination and endoplasmic reticulum-associated degradation (87).

**TABLE 1:**
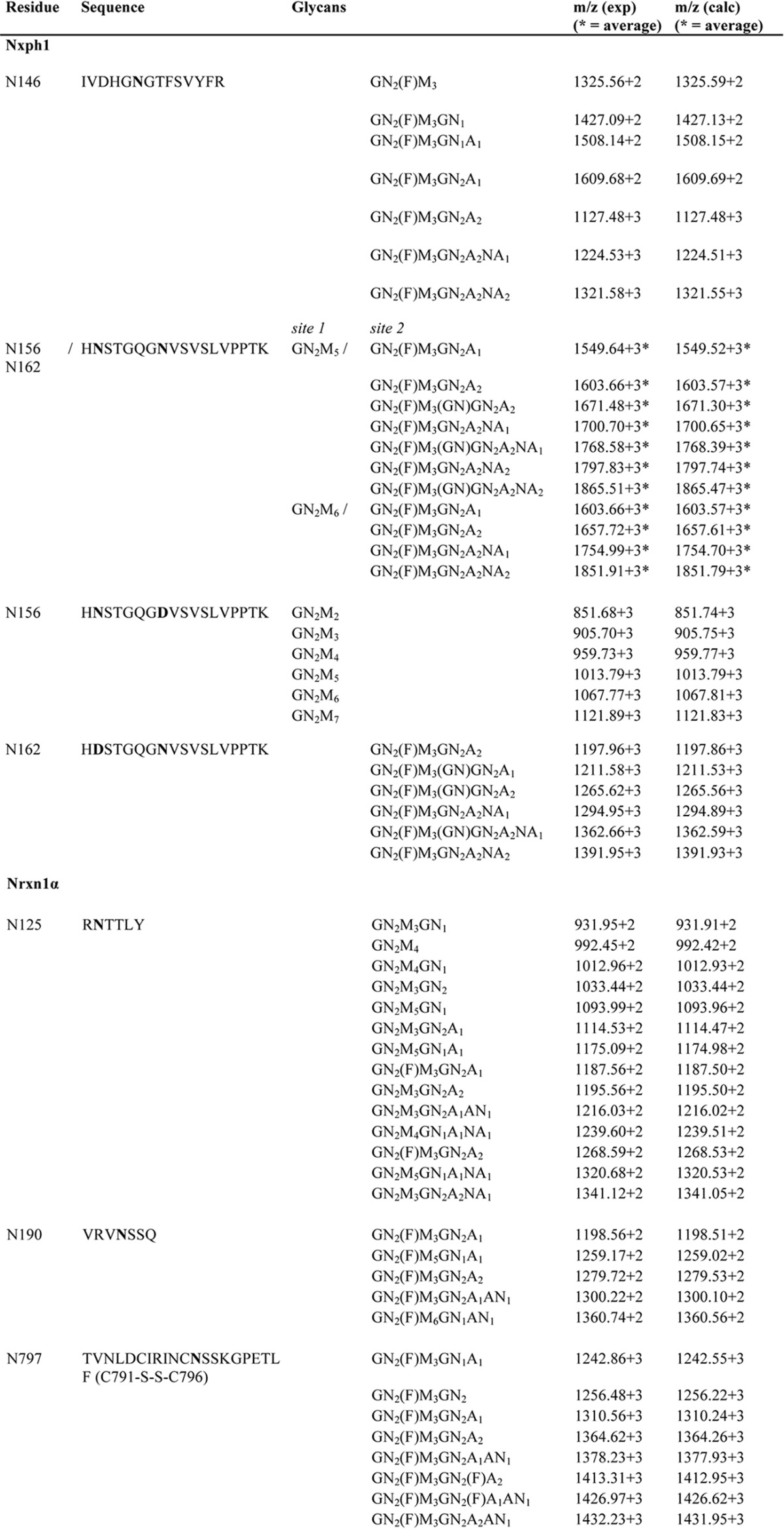
***N-Glycan Structures of Nxph1 and Nrxn1*α** Results are from nanoESI quadrupole time of flight mass spectrometry. Glycans of a complex type were found at Asn-146 and Asn-162 of Nxph1 and at Asn-125, Asn-190 and Asn-797 of Nrxn1α. The high mannose type *N*-glycan exclusively attached to Asn-156 was shown by analyzing wild-type and mutant proteolytic glycopeptides where Asn-162 is inactivated by mutation to Asp. A = Gal, AN = GalNAc, GN = GlcNAc, F = Fuc, M = Man, NA = NeuAc.

##### Determinants of αDAG Binding to α-Nrxn

**FIGURE 5.**
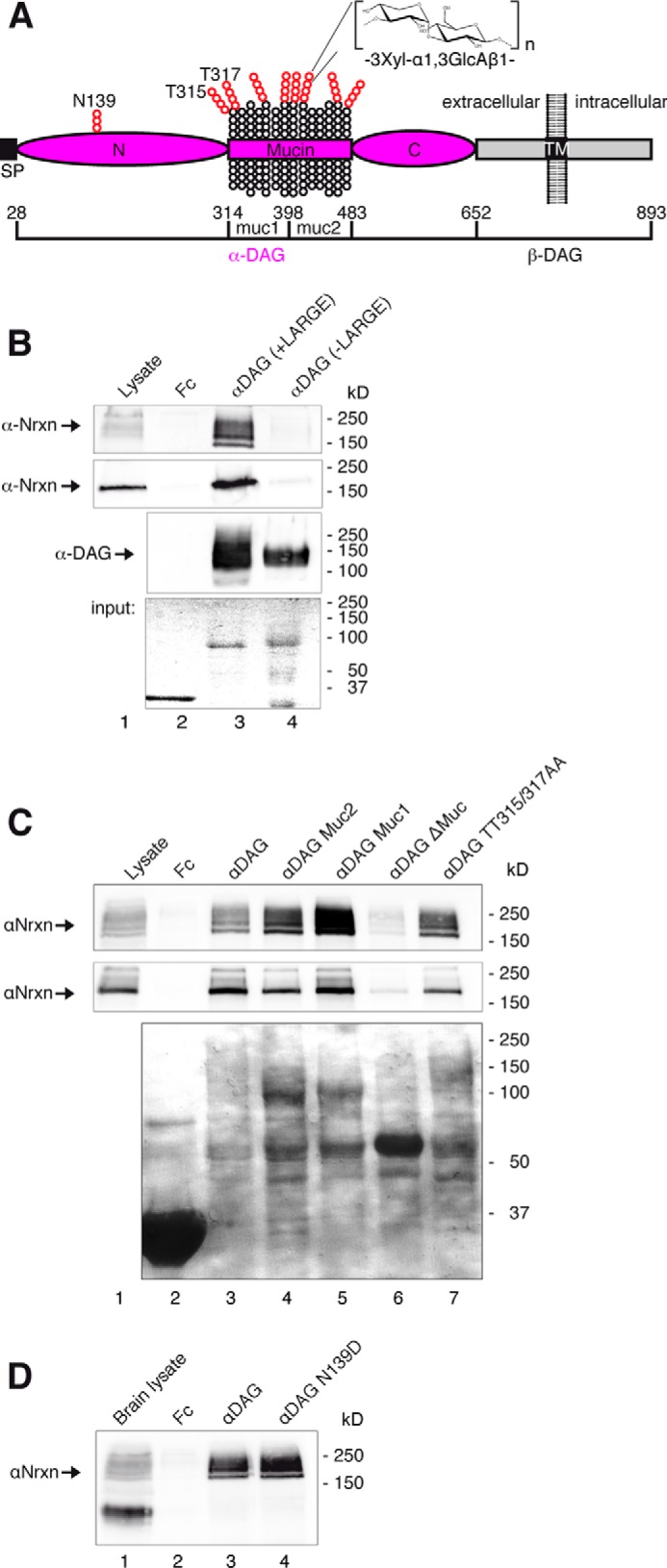
**αDAG binding to α-Nrxn requires LARGE-mediated glycosylation of the mucin-rich region.** *A*, domain structure of DAG with glycosylation sites tested (*red*). *B*, pulldown of α-Nrxn from mouse brain (*lanes 1–4*, *first panel*) or recombinant Nrxn1α from COS-7 lysates (*second panel*) using Fc-tagged αDAG secreted from HEK293 cells. αDAG was co-transfected with (*lane 3*) or without LARGE (*lane 4*); α-Nrxn binds only in the presence of glycans added by LARGE (*lane 3*). *C*, αDAG glycosylation sites essential for laminin binding (*A*, Thr-315, Thr-317; Ref. 90) are not required for Nrxn1α; WT (*lane 3*) and Thr-to-Ala-mutated (*lane 7*) αDAG bind endogenous α-Nrxn (*lane 1*, *first panel*) and recombinant Nrxn1α (*second panel*). Pulldown of endogenous α-Nrxn (*first panel*) and recombinant Nrxn1α (*second panel*) is prevented by the absence of the complete mucin region (*A*) of αDAG (*lane 6*). Removal of half a mucin region (*A*, muc1 or muc2) did not abolish Nrxn1α binding (*lanes 4* and *5*). *D*, *N*-glycosylation in the N-terminal domain (*A*, *N139*) is not essential for binding (*lane 4*). Fc-tagged αDAG variants used in *C* and *D* were produced in presence of LARGE.

Although our experiments above were performed with endogenous, glycosylated dystroglycan from brain or N2a cells, purified recombinant αDAG variants are necessary for mutagenesis. Such experiments were difficult because even large amounts of Fc-tagged αDAG secreted from HEK293 cells were hardly detectable by standard antibodies VIA4–1 or IIH6C4, suggesting insufficient or inappropriate glycosylation (data not shown). This situation changed with the identification of LARGE that successively adds disaccharides of xylose-glucosamine terminal to complex *O-*mannosyl glycans of the mucin region (Fig. 5*A*), which are required for αDAG binding to laminin and agrin (88, 89). To test the role of LARGE for Nrxn binding, we co-transfected HEK293 cells with Fc-tagged αDAG and LARGE and found that glycosylation of αDAG by LARGE is sufficient for binding to endogenous and recombinant Nrxn1α (Fig. 5*B*, *lane 3*, *first* and *second panel*). More importantly, using LARGE-modified recombinant αDAG, we were able to pull down α-Nrxn from mouse brain lysates (*lane 3*, *first panel*), an experiment not even reported for the intensely investigated Nlgn. Although dependence of Nrxn binding on LARGE is consistent with binding to laminin, the sites for the LARGE-mediated glycosylation appear different; we investigated by mutagenesis if Nrxn binds to the αDAG region including Thr-315 and Thr-317 that mediate laminin binding (90) but noticed that the αDAG·Nrxn1α complex formation is not reduced if this region is mutated (Fig. 5*C*, *lane 7*, *first* and *second panel*).

To determine the glycosylated region of αDAG that is responsible for Nrxn binding, we tested the complete and either half of the mucin region (*Mucin*, *muc1*, and *muc2* in Fig. 5*A*). We observed that either half of the mucin region is sufficient to precipitate brain or recombinant α-Nrxn (Fig. 5*C*, *lanes 4* and *5*). In contrast, deletion of the complete mucin region abolished Nrxn binding (*lane 6*). A control mutation of the single *N*-glycosylation site Asn-139 in the N-terminal domain of αDAG had no effect (Fig. 5*D*, *lane 4*). These data indicate that binding of Nrxn to αDAG is locally less restricted compared with laminin, which binds mainly to a distinct N-terminal part of the mucin region (52, 90, 91).

##### Multiplexes of Nrxn1α

**FIGURE 6.**
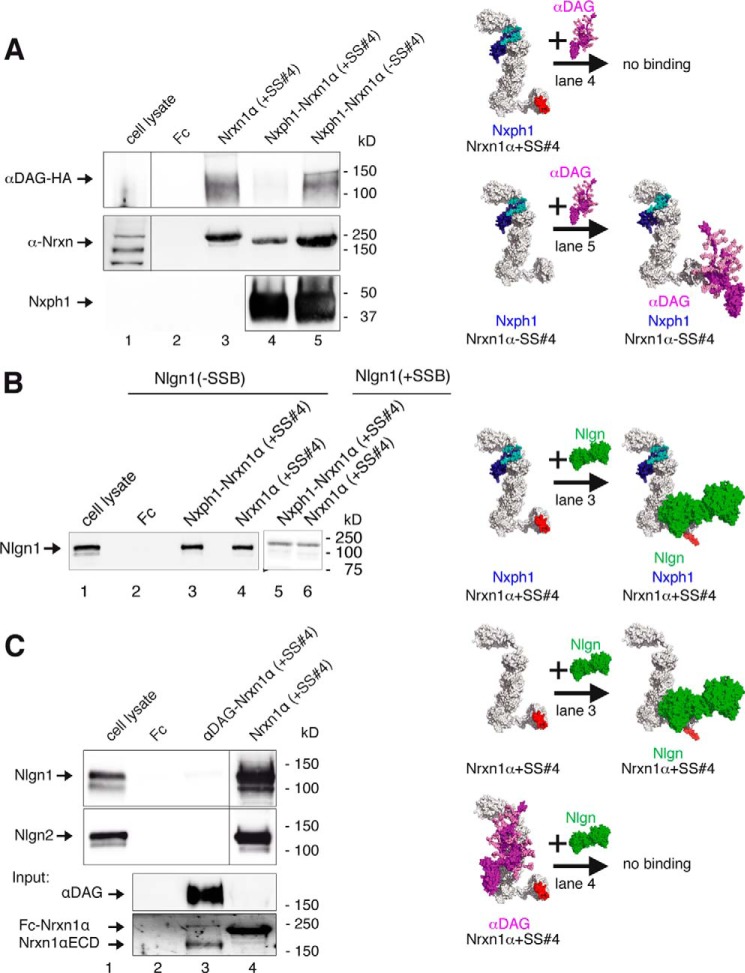
**Restrictions in α-Nrxn multiplexes with αDAG, Nxph1, and Nlgn.** *A*, Nrxn1α triple complex with Nxph1 and αDAG depends on SS#4. Preformed complexes of Fc-tagged Nxph1 and Nrxn1α purified from HEK293 cell media were probed for binding of αDAG-HA from COS-7 lysate with (immunoblot, *lane 4*) and without (*lane 5*) insert at SS#4. A triple complex of αDAG·Nxph1·Nrxn1α only forms with LNS6(−SS#4) (see also Fig. 2*B* and Ref. 37). *B*, triple complex formation of Nlgn1·Nxph1·Nrxn1α has no splicing restrictions. Preformed complex of Fc-tagged Nxph1 and Nrxn1α binds to recombinant Nlgn1 (*lane 3*) even with inserts in Nrxn1α (+*SS#4*) and in Nlgn1(+*SSB*) (*lane 5*), similar to Nrxn1α alone (*lanes 4* and *6*). Binding of Nrxn1α+SS#4 to Nlgn1+SSB requires long incubation and exposure times (20, 25). *C*, αDAG and Nlgns do not bind simultaneously to Nrxn1α. Recombinant Nlgn1(−SSB) and Nlgn2 (*upper* and *middle panels*) bind to Fc-tagged Nrxn1α(+SS#4) (*lane 4*), but a preformed complex of αDAG with Nrxn1α (*lane 3*, *lower panels*) inhibits binding of Nlgn1(−SSB) or Nlgn2 (*lane 3*). All recombinant αDAG variants were purified from HEK293 cells co-transfected with LARGE; pictograms (*A-C*, *right*) visualize the complexes tested and key results (color-coded as labeled, splice inserts are in *red*). For αDAG representation, the model of De Rosa *et al.* (63) was extended with an elongated mucin region, and some recently determined *O-*linked glycans (100) were added. The size of molecules (*right*) are to scale, and the modeled complexes were generated using two criteria, (i) coverage of hot spots and (ii) maximal surface area buried (see “Experimental Procedures”). Note that in addition to complexes shown, other conformations are not excluded. The model of Nlgn/Nrxn1α was modified from Refs. 33 and 66.

The mutually exclusive binding of Nxph1 and αDAG to LNS2 of α-Nrxn shown here indicates that multiple ligand interactions have to be considered to understand the behavior of Nrxn-based molecular complexes. Based on the u-form conformation of α-Nrxn recently identified (Fig. 2*A*), we asked if ligands of the LNS2 and LNS6 domains may influence each other. We found that Nxph1 in complex with full-length Nrxn1α at LNS2 prevents successive binding of αDAG because binding to its second site on the LNS6 is blocked by the presence of an insert at SS#4 (Fig. 6*A*, *lane 4*). A triple complex of Nxph1·Nrxn1α·αDAG is possible, however, when the insert is missing (*lane 5*). Interestingly, binding of αDAG to LNS2 of Nxph1-free Nrxn1α(+SS#4) (*lane 3*) or to LNS6 of Nxph1·Nrxn1α(−SS#4) (*lane 5*) is similarly efficient, indicating an undisturbed binding of αDAG to LNS6 when Nxph1 is associated with LNS2. To brace against artifacts from a mixture of α-Nrxn with and without Nxph1 in these pulldown assays, we used Fc-tagged Nxph1 for a 1:1 stoichiometry with complexed α-Nrxn. After purification, Nxph1 is present as shown by anti-Nxph1 (*lanes 4* and *5*), and the complex was used to test the binding to the third ligand αDAG (Fig. 6*A*, *pictograms* in the *right panel*).

To address a second multiplex, we probed the binding of Nlgn1 to a preformed Nxph1·Nrxn1α complex (Fig. 6*B*). We observed that Nlgn1 binds normally to its sole interaction site at LNS6 independent of the presence of Nxph1 on LNS2 (Fig. 6*B*, *lanes 3* and *4*), resulting into a Nxph1·Nrxn1α·Nlgn1 triple complex (Fig. 6*B*, *right panel*). To analyze the influence of Nxph1 even on residual low affinity binding of Nlgn1 to LNS6, we also included the interaction of Nlgn1(+SSB) to Nrxn1α(+SS#4), a pairing of variants that can barely be detected after long incubation times (*lane 6*) and is even ineffective in synapse formation assays (25, 31). Despite this weak interaction, the Nxph1·Nrxn1α·Nlgn1 triple complex could form in the presence of both splice inserts (*lane 5*), suggesting that interactions of Nxph1 and Nlgn1 with α-Nrxn occur independently of each other, in contrast to αDAG.

Finally, we examined simultaneous binding of α-Nrxn to Nlgns and αDAG. This is an important triple complex because αDAG was reported to mainly localize to inhibitory synapses (45), similar to the Nlgn2 variant (47) that can form a physiologically relevant trans-synaptic complex with α-Nrxn at this synapse (10, 92, 93). We, therefore, included Nlgn2 along with Nlgn1(−B) in our experiments and noticed that both bind equally to Nrxn1α(+SS#4) (Fig. 6*C*, *lane 4*), consistent with a recent SPR study using an isolated LNS6 domain (71). Surprisingly, a preformed complex of Fc-tagged αDAG with Nrxn1α(+SS#4) in which αDAG can only be bound to LNS2 prevented any detectable binding of Nlgn1 or Nlgn2 (Fig. 6*C*, *lane 3*). In support, the reverse experiment with Fc-tagged Nlgn(−B) bound to Nrxn1α(+SS#4) also failed to precipitate αDAG (data not shown), suggesting that a triple complex of α-Nrxn·Nlgns/αDAG is unlikely to occur in brain. This is an unexpected result because αDAG and Nlgn compete in binding to α-Nrxn at different domains. However, it might be explained by the u-form of α-Nrxn that brings binding sites on LNS2 and LNS6 very close to each other (Fig. 2*A*). Thus, the relatively large and similar size of αDAG and Nlgn dimers (Fig. 6*C*, molecules shown in the pictograms are to scale) might sterically hinder each other when bound to LNS2 and LNS6, respectively.

##### Binding Site of αDAG on LNS6

**FIGURE 7.**
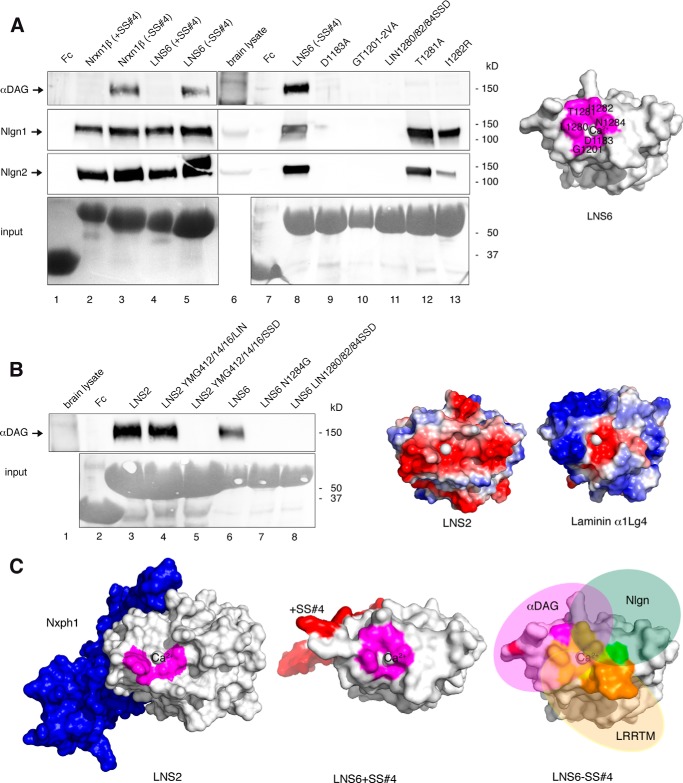
**αDAG competes with Nlgn for binding at Nrxn LNS6 domain.** *A*, site-directed mutagenesis probing the binding epitope of αDAG at LNS6. The presence of splice insert +SS#4 in Fc-tagged Nrxn1β (*lane 2*) or isolated LNS6 (*lane 4*) and two point mutations in LNS6 (T1281A, *lane 12*; I1282R, *lane 13*) selectively block interaction with αDAG from brain lysate (*upper panel*) with unchanged Nlgn1 (*second panel*) and Nlgn2 (*third panel*) binding. Other residues at the calcium coordination site block binding to both αDAG and Nlgn (*lanes 9–11*), resulting in a partially overlapping epitope on LNS6 (*magenta*, *right panel*). *B*, αDAG binding requires calcium-coordinating and hydrophobic residues. Brain αDAG binds to normal LNS2 (*lane 3*) and a hybrid LNS with a triple mutation transferring the hydrophobic calcium coordination of LNS6 onto LNS2 (*lane 4*). Calcium coordination sites transferred from LNS2 to LNS6 (*lane 7*) or from laminin α2LG5 to LNS2 (*lane 5*) or to LNS6 (*lane 8*) abolish αDAG binding. The rim surface of Nrxn LNS2 domain is negatively charged (*red*, *right panel*), in contrast to laminin with more basic residues (*blue*, *right panel*). *C*, structural determinants of Nrxn-ligand interaction. Molecular modeling indicating that binding of Nxph1 at LNS2 (*blue*, *left panel*) and inclusion of splice insert at SS#4 in LNS6 (*red*, *middle panel*) may have a similar structural effect by sterically inhibiting the approximation of αDAG (*right panel*). In addition, binding epitopes for Nlgn (*green*), αDAG (*magenta*), and LRRTM (*orange*) overlap on LNS6 (*yellow*) but also have exclusive residues (*right panel*).

Because the u-form conformation of α-Nrxn allows αDAG to bind simultaneously to LNS2 and LNS6, we finally analyzed the binding epitope of αDAG on the LNS6 domain. To determine the αDAG·LNS6 interface, we first confirmed the dependence of αDAG on alternative splicing in SS#4 as suggested by Sugita *et al.* (Ref. 37 but see Refs. 10 and 25) for different results). In our experiments αDAG binds to Nrxn1β and LNS6 without insert (−SS#4; Fig. 7*A*, *lanes 3* and *5*, *first panel*), and inclusion (+SS#4) completely blocks this interaction (Fig. 7*A*, *lanes 2* and *4*, *first panel*). Because the binding is also calcium-dependent (37), we could successfully abolish the interaction by alanine mutations of the calcium-coordinating residues Asp-1183 (*lane 9*, *first panel*) and Gly-1201 (*lane 10*). As Leu-1280 and Ile-1282 were identified as hot spot residues at the interface of the Nlgn1·LNS6 complex (15–17, 20, 94), we introduced a triple mutation L1280S/I1282S/N1284D that removes hydrophobicity from the surface surrounding the calcium coordination site and observed that it prevents both Nlgn1 and αDAG binding to LNS6 (*lane 11*). This result surprisingly indicates that hydrophobic residues are essential for αDAG. More importantly, we identified an arginine mutation of Ile-1282 that is able to discriminate between Nlgn and αDAG binding to LNS6 by blocking αDAG and leaving Nlgn1 and Nlgn2 unscathed (*lane 13*). These findings suggest that the more limited hydrophobicity of arginine side chains is sufficient for Nlgn association but abolishes αDAG binding. In addition to these overlapping residues, we also discovered that residue Thr-1281 is an exclusive hot spot for αDAG binding not shared by Nlgn (*lane 12*). Together, our results reveal that the binding epitope for αDAG on LNS6 completely circles the calcium binding site (Fig. 7*A*, *right panel*) but also raises the question of how the different epitopes of αDAG on LNS2, LNS6, and on laminin LNS domains relate to each other.

To directly compare binding preferences of αDAG at the two α-Nrxn and the laminin LNS domains (51, 73), we took advantage of their conserved rigid fold (20). We generated hybrid constructs of LNS2, LNS6, and LAMα2LNS5 that express swapped calcium coordination sites and found that the calcium coordination of LNS6 could be transferred to LNS2 with intact αDAG binding (Fig. 7*B*, *lane 4*) but not vice versa (*lane 7*). The laminin calcium coordination contains two serines (PDB code 1QU0) but cannot support αDAG binding when transferred to LNS2 or LNS6 (*lanes 5* and *8*) even with conserved calcium binding to such a hybrid (20). These data suggest that binding of αDAG to LNS domains of Nrxn1α is structurally different to interaction with laminin. This conclusion is supported by the fact that the entire surface of laminin LNS domains is positively charged except for the calcium binding groove (Fig. 7*B*, *right model*) and requires basic residues (51, 73, 74, 95). In contrast, the rim surfaces of LNS2 (Fig. 7*B*, *left model*) and LNS6 (not shown) are mostly negatively charged and display hydrophobic residues near the calcium binding groove. This unusual hydrophobic property of the Nrxn LNS domains was recognized to serve as the LNS6·Nlgn1 interface (15–17, 20, 70) but also mediates the important interaction of αDAG to α-Nrxn as shown above. Comparison of the two αDAG sites on LNS2 and LNS6 revealed that binding of Nxph1 might sterically hinder the approximation of αDAG to the calcium binding site (Fig. 7*C*, *left model*). Interestingly, the insert in SS#4 in switched conformation (71) of the LNS6 (*middle model*) is located at the same side where Nxph1 associates with LNS2, possibly mimicking a similar steric block. In both cases, the binding obstacle may hinder attachment of αDAG to the side near β10-strand of the respective LNS domain (*right model*).

## DISCUSSION

This work presents the first biochemical and structural analysis of binding interference in α-Nrxn-based complexes and identifies important determinants for competition in multiple interactions with Nlgn, αDAG, and Nxph.

### 

#### 

##### Technical Considerations

To study the α-Nrxn multiplexes, we improved methods to isolate and purify αDAG and Nxph1. First, DAG is expressed in most tissues including brain (96), but only αDAG glycosylated by LARGE is able to bind to laminins (97) and Nrxn1α (Fig. 5*B*). We modified the method of Sugita *et al.* (37) by omitting preselection with wheat germ agglutinin because this yielded more Nrxn1α binding αDAG. Although wheat germ agglutinin has been successfully applied to characterize αDAG binding to laminin (51, 52, 73, 90, 97), laminin also precipitates more αDAG without wheat germ agglutinin (98). Second, we observed that neuroblastoma N2a cells are a rich source of Nrxn binding αDAG that facilitated a simple lysis procedures with Triton X-100 as detergent. Third, recombinant Nxph1 has previously been generated by adenovirus-mediated transfection of PC12 cell cultures (35). Here, we developed a less cumbersome alternative strategy by co-expressing Nxph1 with Nrxn1α/LNS2 in HEK293 cells. This procedure yields a high amount of Nxph1·LNS2 complex sufficient even for mass spectrometric analysis of glycan moieties and cysteine connectivity. With these improved tools, we studied Nrxn1α forming binary and tertiary complexes with Fc-tagged αDAG, Nxph1, and Nlgns using a strategy that we successfully applied to determine hot spot residues at the Nrxn·Nlgn interface (20). The results from that previous biochemical investigation were entirely consistent with crystallographic studies (15–17) attesting the reliability of the current approach.

##### Promiscuity of LNS Domains

The calcium coordination site is described as the major binding region in LNS domains for proteins and steroid hormones (19). Our study extends its versatility to binding of glycans as determined here for αDAG·LNS2 and αDAG·LNS6. In addition, our identification of the Nxph1 binding epitope highlights β10 as a second versatile region because the receptor-tyrosine kinase Axl also binds to a corresponding region at LNS1 of Gas6 (75). In support, αDAG likely covers β10 when competing with Nxph1 for binding to LNS2 (Fig. 7*C*), and the insert in SS#4 can replace β10 of LNS6 (71).

It is remarkable that all known, structurally diverse Nrxn ligands bind solely to the LNS2 and/or LNS6 domains. Even more astonishing is the observation that Nlgn, LRRTM, and αDAG may compete for the same epitope at LNS6 (Fig. 7*C*). The reason may reside in an unusual calcium-induced hydrophobic and water-layered interface as shown for Nlgn1·LNS6 (15–17, 20). Such hydrophobic environments are actually predicted to make stable connections to structurally diverse ligands through dynamic variations of hydrophobic contact points (99). Consistently, the conserved interfaces of Nlgn1·LNS6 (PDB codes 3WKF and 3B3Q) and Nlgn4·LNS6 (PDB code 2XB6) differ in their hydrophobic contact points. Moreover, we show here that αDAG also requires hydrophobic residues in the vicinity of the calcium binding sites of LNS2 and LNS6 (Figs. 2*E* and 7*A*).

Because Nrxn binds to complex *O-*mannose-type glycans that completely cover the mucin region of αDAG (Fig. 5*C*) (100–103), it is likely that LNS domains bind only to the accessible sugars but not to protein residues of αDAG. Accordingly, the DAG sequence is highly conserved, and ligand specificity derives from post-translational glycosylation (49). We report here that Nrxn binding depends on LARGE that adds multiple xylose-glucosamine saccharides to glycans attached to αDAG. It is still unknown how exactly sugars bind to LNS domains, but hydrophobic residues are commonly found to participate in binding oligosaccharides (104, 105), and their contribution for stability of a protein·glycan complex can be considerable (106). This idea is supported by crystal structures in which mannose (PDB code 1KZA) or galactose (PDB code 1TLG) coordinate a calcium ion and alkyl or aromatic side chains, performing a hydrophobic stacking with the hydrophobic “bottom” of a sugar pyranose ring (104). Consistently, we determined Tyr-1281 at LNS2 (Fig. 2*E*) and Leu-1280 and Ile-1282 at LNS6 (Fig. 7*A*) as glycan interacting residues.

LNS2 and LNS6 are the only LNS of α-Nrxn with a hydrophobic calcium coordination site. In analogy, αDAG binds solely to the second LNS of pikachurin that has a phenylalanine at the position corresponding to Tyr-412 of LNS2 in Nrxn1α (107, 108). In contrast to these hydrophobic LNS domains, the binding of αDAG to laminin is mediated by basic residues on LAMA2 LG4–5 and LAMA1 LG4 (51, 73, 74, 95). This positive surface fits to the finding that negatively charged sulfated-glycans like heparan sulfate bind to laminin LNS (51, 73, 74, 95). Furthermore, a crystal structure of LAMα2LNS5 (PDB code 1QU0) revealed that a sulfate ion bound to calcium and the αDAG N-terminal mucin region contains sulfated (91) in addition to phosphorylated glycans (52, 109), which explains why laminin binding is restricted to this region (52, 90, 91). In contrast, Nrxn1α also interacts with the C-terminal mucin region (Fig. 5*C*) that is free of sulfated glycans (91). Together, these data allow the definition of two classes of αDAG binding epitopes in the vicinity to calcium coordination sites: (i) a basic surface binding to sulfated or phosphorylated glycans and (ii) a hydrophobic surface binding directly to the pyranose ring of sugars. Although laminin and agrin belong to the first class, pikachurin and Nrxn fall into the second class that is related to C-type carbohydrate sites of lectins, including the αDAG binding concanavalin A (110).

##### Multiplexes of α-Nrxn

**FIGURE 8.**
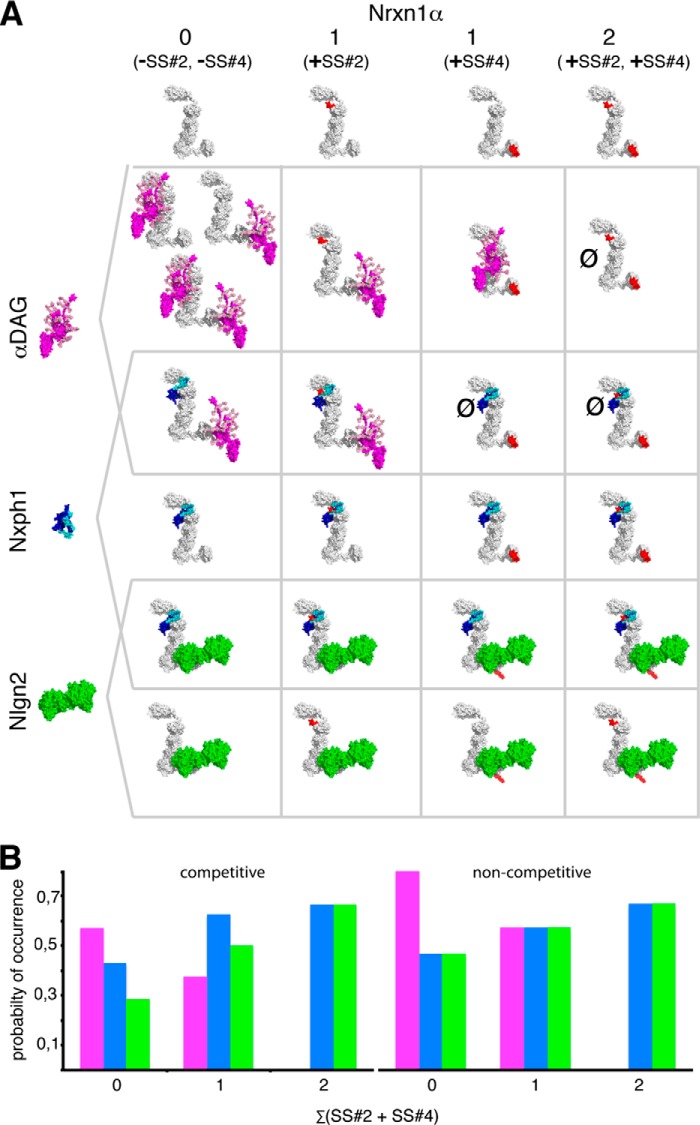
**Schematic summary of α-Nrxn-based multiplexes at a virtual inhibitory synapse.** *A*, binding matrix of αDAG (*magenta*), Nxph1 (*blue*), and Nlgn2 (*green*) forming binary and triple complexes with Nrxn1α (*white*) as determined experimentally in this study. All of these molecules are present or even enriched at inhibitory terminals (Refs. 10, 36, 45 and^47^ and Fig. 1). We used available crystal structures of Nrxn1α (PDB codes 3POY and 3R05) and Nlgn2 (PDB code 3BL8) and generated model structures of αDAG without the N-terminal domain (97) and of Nxph1 to create complexes. The Nlgn dimer and αDAG are of comparable size (radius of gyration (Rg) of 40 and 35 Å, respectively), and both are as long as the rigid core unit LNS2-to-LNS5 of α-Nrxn (Rg = 37 Å). Both αDAG and Nlgn2 cover the LNS5 that may cause steric hindrance and explain the mutually exclusive binding of αDAG and Nlgn2 to Nrxn1α. *B*, probability of Nrxn1α in complex with αDAG (*magenta*), Nxph1 (*blue*), and/or Nlgn2 (*green*), calculated from the total number of possible complexes that are limited by competitive binding (*left panel*). αDAG complexes appear with higher probability when Nrxn1α is splice insert-free, whereas Nlgn2 complexes increase with inclusion of inserts at SS#2 and/or SS#4. If non-competitive binding was assumed, the splice-insert dependence of Nlgn2 and the advantage of αDAG over Nlgn2 complexes would be diminished (no competition, *right panel*). The size of molecules in *A* are to scale, and the modeled complexes were generated using two criteria, (i) coverage of hot spots and (ii) maximal surface area buried (see “Experimental Procedures”). Note that in addition to the complexes shown, other conformations are not excluded. The model of Nlgn·Nrxn1α modified from Refs. 33 and 66.

We tested biochemically the capability of α-Nrxn to form simultaneously complexes (“multiplex”) with its ligands Nxph1, αDAG, and Nlgn. For the purpose of a comprehensive discussion, all possible and “forbidden” multiplexes of α-Nrxns are schematically displayed in Fig. 8*A*. The summary shows, for example, that there are (i) no complexes with both αDAG and Nlgn and (ii) no complexes with αDAG when SS#2 and SS#4 contain inserts, and (iii) all triple complexes include Nxph1. Importantly, all Nrxn1α interactions with αDAG, Nlgn, or Nxph1 investigated here represent irreversible complexes under physiological conditions. Nrxn1α bound to αDAG or Nlgn can only be disassembled by removal of calcium with EDTA (26, 37), whereas Nxph1 can only be dissociated from Nrxn1α by near-denaturing conditions (Fig. 4*D*) (34, 36). As a consequence, disassembly of these complexes needs stringent measures, for example, extracellular metalloproteases that have been shown to cleave βDAG (111), Nlgn1 (112), and Nrxn1α (113) from their membrane-bound C terminus.

Under the simplistic assumption that Nrxn1α ligands are abundantly available and binding occurs randomly, the probability of complex formation is determined by the number of particular ligand complexes divided by all possible complexes. We have analyzed this probability for Nrxn1α complexes with αDAG (Fig. 8*B*, *magenta*), Nxph1 (*blue*), and Nlgn2 (*green*) at a “virtual” inhibitory terminal because these proteins are present at those synapses (45, 47) (Fig. 1). Because the probability depends on the number of relevant splice sites with insert (0, 1, or 2; at SS#2 and SS#4 together), the analysis reveals that αDAG in complex with splice insert-free α-Nrxn forms with a probability of nearly 0.6 (Fig. 8*B*, *magenta*, *left panel*). Nlgn2 complexes (*green*) reach the same value when α-Nrxn contains both inserts, preventing αDAG binding. These individual probabilities of α-Nrxn complexes are a result of the competitive binding that we have biochemically determined for αDAG, Nxph1, and Nlgn. If non-competitive binding was assumed, the probability of an αDAG·α-Nrxn complex without splice inserts would be increased to ∼0.8 (Fig. 8*B*, *magenta*, *right panel*), whereas Nlgn2·α-Nrxn complexes would not change much (*green*, *right panel*). Consequently, our analysis shows that the presence of αDAG can change the probability of Nlgn2·α-Nrxn complexes.

αDAG is expressed much earlier in development than Nrxn1α (114, 115), suggesting that αDAG is an early binding partner. Our hypothesis that the probability of an αDAG·Nrxn1α complex is higher when Nrxn1α carries no splice inserts (Fig. 8*B*) may have important functional implications because some studies indicate that juvenile neurons express mostly insert-negative Nrxn(−SS#4) variants (116).^3^ Moreover, the number of insert-positive variants appears to increase with synapse maturation (116), and +SS#4 expression is reduced after applying a learning and memory paradigm (117). In contrast to αDAG, Nlgn has a higher probability of interacting with Nrxn1α-containing inserts (Fig. 8*B*). Consistent with a role in later developmental stages, there is evidence that synaptic activity and maturation of synapses can increase the insert-positive variants via the calcium/calmodulin-dependent kinase pathway that involves RNA-binding protein SAM68 (24, 118). Thus, developmental and/or activity-regulated control of alternative splicing in Nrxn could modify the composition of multiplexes with αDAG, Nxph1, and Nlgn at inhibitory synapses, adding an exciting and unanticipated layer of complexity to the regulation of these essential molecules.

## Footnotes

3 L. Dudanova and M. Missler, unpublished observations.

## Footnotes

2 The abbreviations used are:

Nrxn: neurexins
SS: splice site(s)
LNS: laminin-neurexin-sex hormone binding
Nlgn: neuroligin
Nxph: neurexophilin
DAG: dystroglycan
LRR: leucine-rich repeat
LARGE: like-acetylglucosaminyltransferase
PAF: penicillium antifungal protein
SPR: surface plasmon resonance
nanoESI: nanoelectrospray ionization.
